# Food-Grade Emulsion Gels as Nutrient Delivery Systems—Standardized Workflow for Fabrication, Characterization, and Application

**DOI:** 10.3390/gels12040298

**Published:** 2026-04-01

**Authors:** Sisheng Li, Minna Luo, Adrian Bogdan Boldianu, David Julian McClements

**Affiliations:** Biopolymers and Colloids Laboratory, Department of Food Science, University of Massachusetts, Amherst, MA 01003, USA; 20201555lss@gmail.com (S.L.); minnaluofood@gmail.com (M.L.); adi.boldianu@gmail.com (A.B.B.)

**Keywords:** emulsion gels, nutrient delivery, nutraceuticals, standardization, bioavailability

## Abstract

Food-grade emulsion gels are increasingly being used to create food products with innovative properties and functional attributes. However, the rapid expansion of research in this area has outpaced the establishment of standardized methodologies, leading to challenges in reproducibility and cross-study comparability. This review addresses this critical gap by providing a comprehensive set of methodological guidelines for the reliable preparation, characterization, and evaluation of food-grade emulsion gels intended for gastrointestinal-targeted nutrient delivery. Initially, systematic approaches for emulsion gel preparation are reviewed, focusing on formulation parameters and processing conditions that dictate the structure and function of these products. A multi-scale framework for physicochemical characterization of emulsion gels is then presented, encompassing structural, rheological, mechanical, thermal, and fluid-holding properties. Guidelines for testing the performance of emulsion gels under simulated food matrix and storage conditions are then given, including methods to monitor bioactive degradation. Furthermore, best practices for evaluating the gastrointestinal behavior of emulsion gels using standardized in vitro digestion models, and subsequent biological evaluation using cell-based assays, animal models, and human trials are discussed. This review concludes that standardized fabrication, characterization, digestion, and reporting protocols are critical for improving reproducibility and comparability across studies and for advancing food-grade emulsion gels toward reliable functional food applications.

## 1. Introduction

Food-grade emulsion gels are versatile colloidal systems that combine the beneficial attributes of emulsions and hydrogels [1]. Typically, they consist of emulsifier-coated oil droplets trapped within an aqueous phase containing a biopolymer network. Emulsion gels can be designed to encapsulate and protect sensitive nutrients during food processing, storage, and gastrointestinal transit, and then release them at targeted rates in the intestine [2]. Consequently, they are highly attractive as nutrient and nutraceutical delivery vehicles in functional food applications, especially when semi-solid or solid products are required. The growing interest in emulsion gels for food applications is reflected in the marked increase in publications in this area over recent decades, as indicated by representative literature database searches. However, the rapid expansion of research on emulsion gels has outpaced methodological standardization, leading to inconsistencies in preparation techniques and evaluation methods across studies. This inconsistency is not unique to the field of food emulsion gels but reflects a broader challenge across modern scientific research. As reported in a *Nature* article, over 70% of researchers had attempted and failed to reproduce the results of another experiment, and more than half had failed to reproduce their own experiments [3]. There are several reasons for this phenomenon, including imprecise or incomplete reporting of the materials and methods used, problems with data processing and reporting, and variations in the procedures used between different studies.

Several review articles have addressed important aspects of emulsion gel research, including structure and rheology, preparation methods, structure–property relationships, and the development of protein- and/or polysaccharide-based emulsion gel systems for food applications and bioactive delivery [1,4,5]. However, these reviews have mainly focused on material design, gelation mechanisms, or selected applications. In contrast, the present review emphasizes methodological standardization and proposes an integrated workflow covering formulation, fabrication, characterization, and reporting considerations for food-grade emulsion gels as nutrient delivery systems.

**Figure 1:**
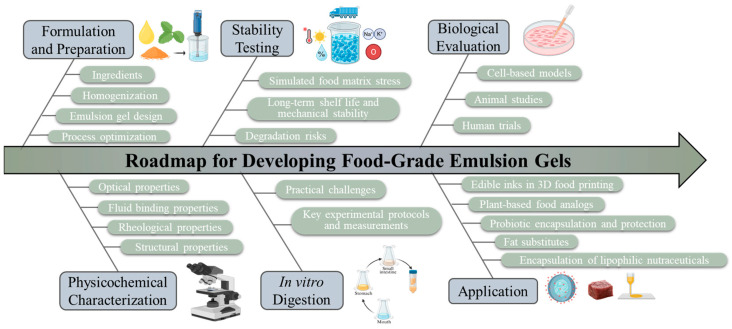
Workflow roadmap for developing food-grade emulsion gels.

Accordingly, this review presents a systematic, end-to-end framework for the formulation, characterization, and biological validation of food-grade emulsion gels (Figure 1), while critically examining how formulation influences the bioaccessibility, bioavailability, bioactivity, and health effects of encapsulated compounds. By analyzing current practices, identifying gaps in methodological consistency, and proposing clear reporting recommendations (Table 1), this article provides a practical guide for evaluating emulsion gels from initial design through digestion-related performance (Table 2). This framework is intended to improve reproducibility and comparability across studies, support more reliable bioactive delivery, and accelerate the development of healthier functional foods. In addition, high-quality and consistent datasets generated using standardized protocols may provide a stronger foundation for future artificial intelligence and machine learning approaches in product development [6,7,8,9].

## 2. Formulation and Preparation

### 2.1. Key Ingredients

#### 2.1.1. Oil Phase

Oils are often selected for their beneficial nutritional attributes, which depend on their fatty acid profiles, as well as the presence of minor bioactive components [34,35]. Polyunsaturated oils, especially those rich in omega-3, offer health benefits but oxidize quickly, so protective packaging, storage conditions, and antioxidant strategies are required to protect any emulsion gels fortified with polyunsaturated fatty acids [35,36,37]. Fatty acid chain length and unsaturation also modulate digestion and absorption, with unsaturated species such as linoleic and linolenic acids having enhanced uptakes [38]. Natural minor components such as tocopherols, sterols, oryzanol, and chlorophylls play critical roles in the photostability and oxidative stability of emulsified oils [39,40]. Emulsified oils rich in antioxidant substances (like rice bran oil) have also been shown to exhibit higher cellular antioxidant activity than those that are not [41].

Oil physical properties govern the formation and stability of emulsions. Higher viscosities and interfacial tensions require more homogenization energy and typically lead to larger droplets [42]. Oil polarity governs how emulsifiers adsorb at the oil–water interface, which determines the resistance to coalescence [43]. Moreover, polar oils can dissolve in water, which promotes Ostwald ripening, where larger droplets grow at the expense of smaller ones due to diffusion through the continuous phase [44,45]. This instability mechanism can be retarded or prevented by adding a sufficiently high amount of a ripening inhibitor to the oil phase [46]. Food-grade ripening inhibitors are usually highly hydrophobic substances with lower water solubility, such as long-chain triglyceride oils. Polarity also influences the interfacial conformation of some emulsifiers, which affects emulsion formation and stability [47,48].

Once formed, the density contrast between the oil and water phases will impact the rate of gravitational separation in an emulsion, therefore impacting their long-term stability [49]. A greater density difference leads to faster creaming. This is common with essential and flavor oils that are considerably lighter than water. Formulators can raise the effective density of these oils using permitted weighting agents to reduce creaming during storage [46].

The oil volume fraction (φ) also determines emulsion stability and rheology. At φ < 0.1, the oil droplets in emulsions remain largely isolated from each other (provided they are not strongly attracted to each other), which leads to relatively low viscosity and good flowability. As φ increases, droplet interactions increase and viscosity rises. When the oil phase content approaches the maximum close-packing limit (φ ≈ 0.64–0.74 for monodisperse spheres), the oil droplets cannot easily move past each other, which leads to solid-like characteristics, such as a yield stress and elastic modulus [50,51].

Moreover, the droplet concentration will impact the appearance and texture. Typically, the lightness of an emulsion increases steeply when the oil droplet concentration increases from 0 to 5% and then remains relatively high when the oil droplet concentration is raised further [52]. The mechanical properties of emulsion gels depend on droplet concentration, size, and interfacial properties. Oil droplets may strengthen or weaken a gel depending on whether they act as active fillers that interact strongly with the matrix or as inactive fillers that do not, which determines hardness and fracture behavior [53].

#### 2.1.2. Emulsifier

**Figure 2:**
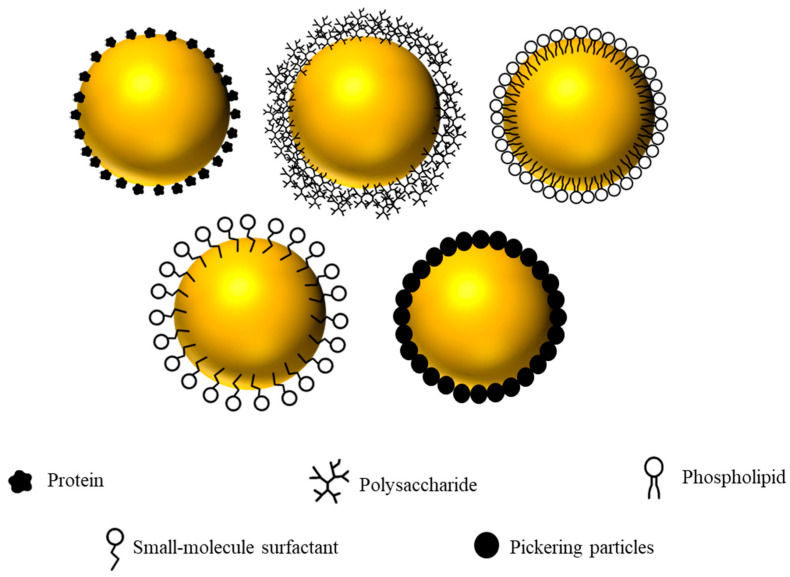
A schematic illustration of five major emulsifier classes at the oil–water interface. The yellow core represents an oil droplet; the surrounding symbols denote different stabilizers.

The type and amount of emulsifiers used are critical for the formation and stabilization of emulsions [46] and therefore play an important role in the production of emulsion gels with the required properties. Emulsifiers lower the oil–water interfacial tension during homogenization, thereby generating smaller droplets, and they form protective interfacial layers that limit aggregation during and after processing. Common food emulsifiers are described below and shown in Figure 2.

*Proteins*. The amphiphilic nature of proteins enables rapid adsorption to oil–water interfaces and the formation of protective interfacial layers that inhibit droplet aggregation through electrostatic and steric repulsion [1,54,55]. Traditionally, the animal proteins from egg, meat, and milk were used as emulsifiers in the food industry, which vary in their molecular structures and functionalities. In milk, the whey proteins are relatively small globular proteins, whereas the caseins are intrinsically disordered proteins. Both whey protein- and casein-coated oil droplets are highly susceptible to aggregation at pH values near their isoelectric point and at high ionic strengths due to a reduction in the electrostatic repulsion between them [56,57,58]. Casein-coated droplets typically better withstand thermal processing, while globular whey proteins unfold above their denaturation temperature, exposing hydrophobic and sulfur-containing residues that promote hydrophobic interactions and disulfide bonding. Recently, plant proteins such as soy, pea, and potato proteins have gained interest for ethical and sustainability reasons [59,60]. However, these are globular proteins that show similar aggregation behavior near the isoelectric point, at high ionic strengths, and upon heating.

*Polysaccharides*. They are often used as stabilizers in emulsions because of their ability to greatly increase the viscosity of the aqueous phase [54,55]. Most of these polysaccharides are large hydrophilic molecules and therefore unsuitable as emulsifiers. However, some natural polysaccharides (like gum arabic and beet pectin), as well as some chemically modified polysaccharides (such as octenyl succinic anhydride (OSA) modified starch), do possess amphiphilicity and so can be used as emulsifiers. The non-polar groups on these polysaccharides allow them to adsorb to oil–water interfaces, where they form thick interfacial layers that can stabilize emulsions through a combination of steric and electrostatic repulsion [61]. The main disadvantage of polysaccharide-based emulsifiers is that relatively high emulsifier-to-oil ratios are usually required (>0.5-to −1). In contrast, the main advantage is that they can form oil droplets that are relatively resistant to changes in pH, ionic strength, and temperature because they are mainly stabilized by strong steric repulsion.

*Phospholipids*. They represent a class of natural, low-molecular-weight surfactants that are commonly used as food emulsifiers [54,55]. In nature, phospholipids play a fundamental role in determining the structure and function of biological membranes [62]. The resulting interfacial films protect droplets from aggregation through electrostatic repulsion. Practical limitations include poor water dispersibility, oxidative instability, and compositional heterogeneity. Lysolecithins, produced by removing one acyl tail using hydrolysis, often disperse better in water and show higher interfacial activity, though they may impart off-flavors [63].

*Small-molecule surfactants*. Both natural and synthetic small-molecule surfactants are available for utilization as emulsifiers in the food industry [54,55]. As an example, Tweens and Spans are commonly used examples of synthetic surfactants, whereas quillaja saponin is an example of a natural one. Typically, surfactants can form and stabilize emulsions when used at relatively low emulsifier-to-oil ratios (<0.1-to −1). The type of emulsion they can stabilize depends on their hydrophile–lipophile balance (HLB), with oil-in-water emulsions being formed for relatively high HLB numbers (>10) and water-in-oil emulsions being formed for relatively low HLB numbers (<6) [46]. There are several drawbacks to using synthetic surfactants, including sensory off-notes, regulatory restrictions on permissible food-grade surfactants, gastrointestinal sensitivities in certain consumers, and poor consumer acceptance of synthetic additives [54,55].

*Pickering emulsifiers*. Pickering emulsions are stabilized by solid particles (rather than amphiphilic molecules) that irreversibly adsorb to oil–water interfaces and form robust mechanical barriers that effectively inhibit droplet coalescence [64,65]. For example, pea protein particles have been shown to stabilize mayonnaise-style products with exceptional resilience to changes in pH and increases in ionic strength [66]. Nonetheless, the requirement for higher particle loadings can result in increased turbidity and a potentially gritty mouthfeel.

#### 2.1.3. Gelling Agents

Food-grade gelling agents are also critical components for the creation of emulsion gels with specific functional attributes. The aqueous phase of emulsion gels is gelled by adding biopolymers, such as proteins and/or polysaccharides, that are capable of forming a 3D network through physical or chemical crosslinking [53]. Common mechanisms include heat-, cold-, ion-, enzyme-, and acid-induced gelation [67]. Gelatin and gellan gum form reversible cold-set gels due to coil-to-helix transitions during cooling, which promote the formation of hydrogen bonding between helical regions on different molecules. In contrast, globular proteins, like whey, soy, or pea proteins, form irreversible heat-set gels because they unfold during heating, leading to the exposure of non-polar and sulfur-containing amino acids at their surfaces, which then promote the formation of hydrophobic and disulfide bonds between different molecules. Anionic polysaccharides, such as alginate, carrageenan, gellan gum, and pectin, can form gels in the presence of sufficiently high concentrations of cationic ions (such as Ca^2+^) [68]. Some biopolymers can form acid-induced gels because altering the pH alters the electrostatic repulsion between them. To illustrate, caseinate forms acid-induced gels near its isoelectric point because the reduced electrostatic repulsion promotes network formation [69]. Biopolymers can also be crosslinked using specific kinds of enzymes. Notably, microbial transglutaminase can be used to crosslink proteins by catalyzing the formation of ε-(γ-glutamyl)-lysine bonds, leading to the formation of strong hydrogels [70,71]. Recent reviews outside the food field further highlight the broad translational relevance of biopolymer-based emulsion gels, showing that food-grade proteins and polysaccharides can be used to assemble advanced soft materials with a range of functionalities [72,73].

In general, gel strength and microstructure depend on the type and concentration of biopolymers and gelling agents, and blends can be designed to produce phase-separated, interpenetrating, or co-gelling composites [53,68].

### 2.2. Homogenization Methods

**Figure 3:**
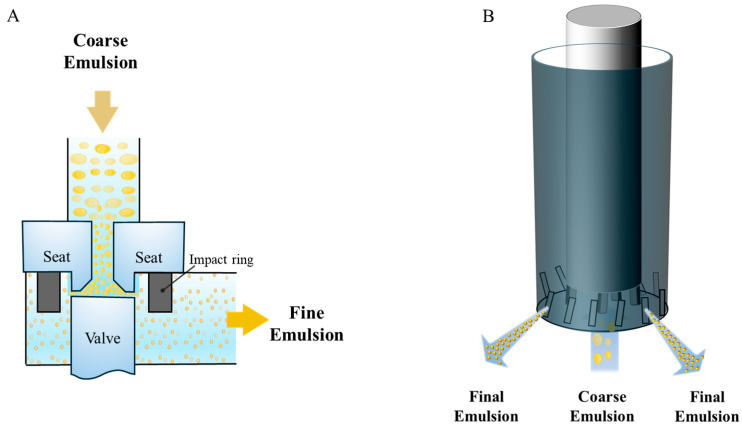
Two homogenization methods. (**A**) High-pressure needle-on-seat valve with reverse-flow for repeated shear cycles. (**B**) Rotor–stator mixer for intense shear in the rotor–stator gap. Illustration created in BioRender.

The type of homogenizer used, as well as the operating conditions employed, impact on the initial droplet size, dispersity, and interfacial properties of emulsions, which in turn govern the structure and functionality of emulsion gels. A variety of different kinds of mechanical homogenizers are available for creating food emulsions, including rotor–stator mixers (RSMs), colloid mills, high-pressure valve homogenizers (HPVHs), sonicators, and microfluidizers, which vary in their costs, scalability, efficiency, and adaptability to diverse formulations [74] (Figure 3).

RSMs and colloid mills can be used to homogenize viscous systems, but they generally produce droplets in the micron range [75]. In RSMs, a high-speed rotor drives turbulent jets through a perforated stator. In colloid mills, a pre-emulsion is sheared in a narrow gap between rapidly moving surfaces. High-pressure valve homogenizers force a pre-emulsion through a narrow valve gap at roughly 10–100 MPa, creating intense turbulence and smaller droplets, commonly 150–500 nm. They work best for low-to-intermediate viscosity fluids. Microfluidization and sonication are frequently used at the lab scale to reach submicron sizes, but can be used commercially [74].

In addition to the type of homogenizer used, the operating conditions are also important [74]. The speed and duration of rotation in RSMs and colloid mills impact the droplet size, whereas the operating pressure and number of passes in HPVHs and microfluidizers impact the droplet size. Typically, the droplet size decreases with increasing energy input, but sometimes overprocessing can adversely affect emulsion formation. Moreover, the sequence of adding gelling agents before or after homogenization should be optimized [76].

### 2.3. Types of Emulsion Gel

#### 2.3.1. Emulsion-Filled Gels

**Figure 4:**
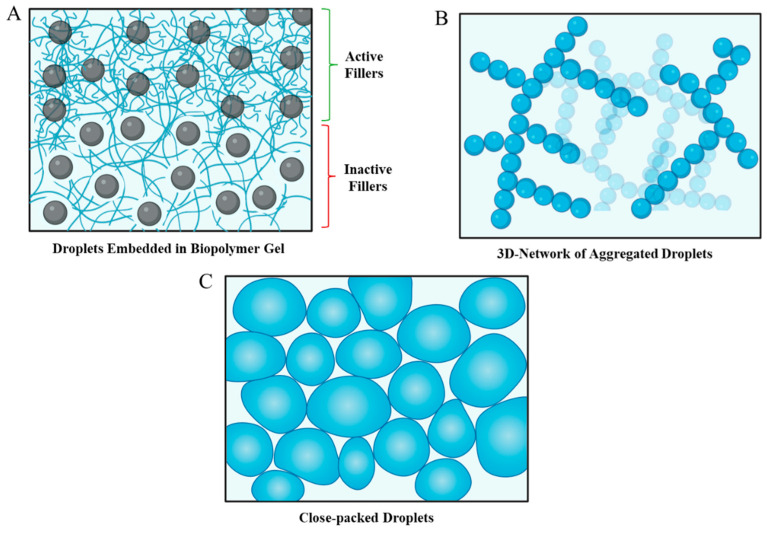
Schematic representation of three classes of emulsion gels. (**A**): Emulsion droplet-filled gel with active fillers (upper) and inactive fillers (lower). (**B**): Emulsion droplet-aggregated gel. (**C**): High internal phase emulsion gel.

Emulsion-filled gels consist of oil droplets embedded within an aqueous phase that contains a biopolymer gelled network (Figure 4A). These complex colloidal systems can be categorized according to their two main features. (1) The gel network—the nature of the biopolymer network surrounding the fillers impacts the overall gel properties, such as the type and concentration of proteins and polysaccharides present, and the crosslinking mechanism, which may be reversible or irreversible, cold-set, heat-set, ion-set, pH-set, or enzyme-set. (2) The filler type—the type, concentration, and interactions of the oil droplets present are also important. The droplets may act as active fillers that directly interact with the surrounding gel network and modify its properties, or they may be inactive fillers that act as inert space-occupying inclusions that have minimal interactions with the surrounding gel network [77].

#### 2.3.2. Droplet-Aggregated Emulsion Gels

In droplet-aggregated emulsion gels (Figure 4B), the oil droplets themselves form the network structure that provides solidity to the whole system. This type of emulsion gel is formed by controlled aggregation of the oil droplets, which can be achieved through various mechanisms, including depletion flocculation, bridging flocculation, pH-induced, ion-induced, heat-induced, or enzyme-induced aggregation [1,2]. In this case, there is therefore no need for a gelling agent within the aqueous phase. Instead, the system conditions are altered to promote the aggregation of the emulsifier-coated oil droplets. In this case, the method used to promote droplet flocculation therefore depends strongly on the nature of the emulsifier used to coat the oil droplets. To illustrate, protein-coated oil droplets can be made to aggregate by adjusting the pH close to the isoelectric point of the proteins, adding salts, or heating above the thermal denaturation temperature of the proteins [78].

Notably, many real-world emulsion gels do not fall neatly into one category or the other, often exhibiting hybrid characteristics of both emulsion-filled and droplet-aggregated systems [79].

#### 2.3.3. High Internal Phase Emulsion (HIPE) Gels

High internal phase emulsions have a dispersed phase volume fraction that typically exceeds about 0.74. As a result, the oil droplets are tightly packed together (“jammed”), which prevents them from easily moving past each other, thereby leading to some elastic-like properties [80] (Figure 4C). Again, no gelling agent is needed to form these emulsions, and the oil droplets do not need to be strongly attracted to each other. However, it is important that the oil droplets are coated by a robust interfacial layer so as to prevent their coalescence, as the droplets are tightly packed together for extended periods, which can cause them to merge together. For this reason, particle-based emulsifiers (Pickering stabilization) are often used to form and stabilize these emulsions because they create interfaces that are highly resistant to coalescence [66].

### 2.4. Practical Aspects to Consider When Preparing Emulsion Gels

#### 2.4.1. Raw Material Consistency

Consistent functional performance of raw materials is important. Natural biopolymers used as emulsifiers or gelling agents can exhibit considerable batch-to-batch variability in terms of their compositions, molecular weight distributions, purities, and degree of chemical modification, which can profoundly impact their emulsifying capacity, gelling kinetics, and final gel strength. Soy protein functionality depends on extraction and processing conditions. Therefore, researchers should specify suppliers and lots, characterize key properties, and report them to enable reproducibility [81].

#### 2.4.2. Homogenization and Gelation Processing Parameters

Robust products require optimized and controlled processing. Mixing order, speed, and time determine initial droplet formation and uniform distribution of oil, biopolymers, and gelling agents. Temperature control influences thermally induced gelation, enzyme activity, and component solubility. The pH and ionic strength must be measured and maintained throughout preparation because they govern charge, conformation, and interactions of emulsifiers and gelling agents [74].

#### 2.4.3. Gelation Kinetics and Emulsion Stability

The structure and properties of emulsion gels depend on a balance between gelation and emulsion destabilization kinetics [1,82]. Unbalanced gelation and emulsion destabilization kinetics can arrest non-equilibrium structures, leading to heterogeneous networks and non-uniform droplet distributions [83]. Under these conditions, the gel often lacks mechanical strength and can separate into different phases.

### 2.5. Recommendations for Emulsion Gel Preparation

It would be advantageous to develop a standardized operating procedure (SOP) that describes the ingredients and processes required for consistent emulsion gel preparation that all personnel can follow. All critical parameters should be controlled and recorded, including ingredient types and concentrations, holding temperatures and times, heating and cooling rates, pH, ionic strength, salt type and concentration, and mixing and homogenization settings conditions with equipment type, geometry, speeds, times, pressures, and number of passes. The exact order of ingredient addition should be specified and kept constant across batches. Well-characterized raw materials should be used, and the supplier, product name, product code, and lot number should be recorded and reported. Where feasible, the characterization of key emulsifiers and biopolymers should be performed and reported, including purity, molecular weight distribution, and, for proteins, the denaturation and aggregation state.

## 3. Characterization of Emulsion Gels

### 3.1. Microstructural Analysis

#### 3.1.1. Confocal Laser Scanning Microscopy (CLSM)

CLSM is a powerful microscopy technique for examining the microstructure of emulsion gels, especially when combined with fluorescence staining [60]. It provides detailed information on the dimensions, location, and aggregation state of oil droplets, as well as the morphology of the surrounding biopolymer network [10]. This is accomplished by using specific fluorescent stains to distinguish between different components like lipids, proteins, and polysaccharides. An advantage of CLSM is its ability to image samples in their native, hydrated state. It offers optical sectioning to create 3D reconstructions, making it ideal for visualizing the structural organization of the different components in emulsion gels.

Despite its strengths, the effective use of CLSM requires careful selection of fluorescent dyes to ensure accurate staining of the intended components without inducing artifacts and triggering structural changes [84,85]. Dyes must be chosen and applied carefully to avoid their partitioning between phases and causing undesired changes in gel structure. Some of the main limitations of this method are photobleaching of dyes, shallow penetration depths in turbid samples, resolution constraints (typically limited to a few hundred nanometers), and challenges in reliably obtaining thin slices of the sample that represent the original material [86].

#### 3.1.2. Electron Microscopy

Electron microscopy techniques offer significantly higher spatial resolution than conventional light microscopy or CLSM, allowing for the detailed examination of gel network ultrastructure, droplet morphology, and interfacial layers [87].

Conventional scanning electron microscopy (SEM) is based on measuring the scattering of electrons from the surfaces of samples under high vacuum. Dehydration and sputter coating of biopolymer gels alter their microstructures, so the images obtained should be interpreted with care. Notably, freeze-drying can distort biopolymer networks, while metal coatings (typically 5–10 nm) can enlarge measured pore sizes by tens of nanometers [11,88]. Nevertheless, SEM remains valuable for analyzing surface topography and network porosity in dried gels [89]. Analysis of freeze-dried soy protein–pectin gels revealed a spheroidal droplet network with coarse, loosely connected pores in untreated samples, while producing a more uniform, dense microstructure with minimal droplet aggregation [90]. A common mistake is to overinterpret or misinterpret scanning electron microscopy images. Researchers sometimes assume that the microstructures seen in the images of freeze-dried samples accurately represent those found in the original sample, which is unlikely, given that freezing and dehydration may alter the network structure.

Cryo-SEM reduces drying artifacts by rapidly freezing hydrated samples and imaging at cryogenic temperatures, which better preserves their original microstructures. Even so, special attention should still be paid to sample preparation for cryo-SEM analysis, since the rapid formation of ice crystals can still compromise the structural integrity of samples unless counteracted by using cryoprotectants or high-pressure freezing techniques [91,92].

In contrast to SEM, transmission electron microscopy (TEM) passes a beam of electrons through a sample to create an image, with the different structures being detected due to differential transmission of electrons through areas in a sample with different electron density. For this reason, reliable results depend on consistent fixation, embedding, sectioning, and staining, and on awareness that drying or staining can introduce contrast that does not fully represent the native state [11].

Careful documentation of all sample preparation procedures is essential, which includes fixation, dehydration, embedding, sectioning, staining, coating, and freezing, as these steps are primary sources of structural artifacts. Regardless of the sample preparation method used, interpretation of microscopy images must always be performed with an awareness of the potential for preparation-induced structures.

#### 3.1.3. Scattering Techniques

*Dynamic light scattering (DLS)*. DLS estimates particle size from time fluctuations in scattered-light intensity caused by Brownian motion of the particles. It is most suitable for diluted dispersions of small particles (3 to 5000 nm) dispersed in low-viscosity Newtonian fluids (like water). It is unsuitable for colloidal dispersions containing larger individual particles or flocculated particles because their slow or constrained motion leads to little or no Brownian motion.

*Static light scattering (SLS)*. SLS is another widely used scattering technique for providing information about the particle size distribution, mean particle diameter, and polydispersity of the emulsions used to prepare emulsion gels [84]. In this case, the intensity of scattered light is measured as a function of the scattering angle. Particle size information can be obtained by analyzing the resulting scattering profile using a suitable mathematical model (such as Mie theory) because the intensity and angle of light scattering depend on particle size. This technique is sensitive to particle sizes between about 100 nm and 2500 nm. SLS instruments are also available that can provide information about the properties of biopolymers in solutions, such as proteins and polysaccharides, including the weight-average molecular weight, radius of gyration, and second virial coefficient. Researchers have shown that variations in the second virial coefficient and molecular weight of caseinate solutions are closely tied to the calcium-induced aggregation and onset of gelation in emulsions [93].

*Small-angle X-ray scattering (SAXS) and small-angle neutron scattering (SANS)*. SAXS and SANS are powerful tools for probing nanoscale structures, typically in the one to several hundred nm range [94,95,96]. These techniques can be used to obtain a wide range of information about emulsions and emulsion gels, including the size, structure, and aggregation state of colloidal particles (such as oil droplets or protein particles), the thickness and packing of the interfacial layer, and the mesh size or correlation length of biopolymer networks in gels. SAXS has been used to determine changes in the structural features of casein gels during digestion, such as aggregate size and packing [97]. An advantage of SANS is that contrast matching of different phases can be achieved through isotopic substitution (e.g., by replacing some or all of the H_2_O in the aqueous phase with D_2_O), which allows specific structural features within complex samples to be characterized, such as interfaces or droplets [98]. Due to their greater penetration depth, SAXS and SANS can be used to characterize solid or semi-solid samples, like the particles in emulsion gels. However, data interpretation requires appropriate structural models, and multiple-scattering corrections may be necessary.

In situ *SAXS/SANS under flow or microfluidic control*. Coupling microfluidics or rheo-scattering with SAXS/SANS enables millisecond, in situ tracking of nanoscale to microscale structures during gel formation. A SAXS experiment at the European Synchrotron Research Facility (ESRF)-ID02 with a microfluidic extrusion device showed that nanoparticles first assemble at the interface and only then form (sub)micron bicontinuous networks during bigel formation [99]. For food matrices, paired SAXS/SANS (with contrast variation) and rheo-SAXS provide in situ access from primary building blocks to particle networks under processing-relevant shear or flow conditions, which enables process–structure–function mapping for emulsion gel design [100]. In practice, these tools help food scientists select the appropriate pH, salt, solvent exchange, and shear conditions to determine particle size and biopolymer network structures.

#### 3.1.4. Emerging High-Resolution Structural Imaging for Emulsion Gels

*Super-resolved vibrational microscopy*. This technology provides intrinsic, label-free chemical contrast that is especially suited to the study of soft matter foods. Stimulated Raman scattering (SRS) delivers high-speed chemical maps with 3D optical sectioning and high sensitivity, enabling quantitative imaging of major food biomolecules without dyes [101]. In polymers and soft materials, anti-Stokes Raman scattering (CARS) produces signals that are much stronger than those obtained by spontaneous Raman scattering. This allows for rapid, label-free compositional imaging and optical sectioning. These capabilities has been used to map lipid–protein–water domains and interfaces in complex emulsion gels [102]. Recent advances break the diffraction limit for coherent vibrational imaging: phase-resolved image-scanning CARS achieves up to a twofold lateral resolution improvement with a simple, low-intensity add-on compatible with conventional forward-detected CARS [103]. Within food analysis, Raman-based methods provide improved sensitivity, real-time performance, and applicability to turbid matrices, underscoring their potential for compositional mapping of semi-solid foods [104].

*Stimulated emission depletion (STED) and fluorescence lifetime imaging microscopy (FLIM) combinations*. Pairing STED with FLIM enables information about microstructures and local physicochemical microenvironments to be obtained in soft solid foods, like emulsion gels. STED provides microstructure analysis beyond the diffraction limit, while FLIM provides microenvironment property information. As an example, acidified skim milk gels containing pectin were imaged using STED and a viscosity-sensitive FLIM rotor probe, revealing pectin-dependent changes in correlation length, void spacing, and lifetime contrasts across protein, interfacial, and void regions [105]. This combined readout can resolve droplet and network contacts, quantify protein network density, and probe interfacial protein conformations.

### 3.2. Physical Properties

#### 3.2.1. Shear Viscosity Analysis

The rheological properties of emulsions and fluid-like emulsion gels can be characterized by measuring their apparent shear viscosity *versus* shear rate (or shear stress) profiles. These measurements are usually carried out using a mechanical viscometer or shear rheometer. Typically, a sample is placed in a suitable measurement cell, such as a concentric cylinder, cone-and-plate, or plate-and-plate system, incubated to the required temperature, and then the shear rate is measured as the shear stress is increased (or vice versa) [106,107]. The resulting shear viscosity *versus* shear rate (or shear stress) profile can then be calculated from this data. For samples that exhibit plastic-like behavior, the yield stress and plastic viscosity can also be calculated from these plots.

#### 3.2.2. Small Amplitude Oscillatory Shear (SAOS) Rheology

SAOS rheology is one of the most common analytical techniques used to characterize the linear viscoelastic behavior of emulsion gels. The small deformations applied to the samples for these measurements do not disrupt their structures and can therefore be considered to be non-destructive. These tests provide information about the fundamental rheological properties of samples, such as the storage modulus (G′), loss modulus (G″), loss tangent (tan δ = G″/G′), and complex shear viscosity (η*), which provide insights into gel strength and the viscoelastic balance (i.e., how solid-like or liquid-like a sample is). Initially, strain (or stress) sweep tests are employed to determine the linear viscoelastic region (LVR) of the material, within which the rheological properties are independent of the applied strain. Within the LVR, the magnitude of G′ reflects the gel strength, with a higher value indicating a harder gel. Frequency sweep tests involve measuring the frequency dependence of the G′ and G″ values (typically from around 0.01 to 100 Hz) of a material, mapping elastic and viscous contributions. Time sweeps involve measuring the change in the G′ and G″ values with time, usually at a constant temperature, strain, and frequency. This type of test can be used to monitor gelation kinetics, with the gel point often being marked as the crossover of the G′ and G″ values [108,109]. Temperature sweeps with controlled heating and cooling profiles capture the setting and melting of heat-set and cold-set gels.

For methodological consistency, it is crucial to report the measurement cell type, geometry, dimensions, temperature control strategy, and how the LVR was determined. Proper loading techniques must also be ensured to prevent damage to samples, edge effects, and wall slip. For fluid samples, it is important to ensure that creaming or sedimentation of any particles does not occur during the measurements, as this will lead to an inhomogeneous sample, thereby leading to unreliable results. During heating, it may also be important to minimize the evaporation of water by using a solvent trap or covering the exposed edges of the sample with oil; otherwise, the rheology of the sample will change, leading to erroneous results.

#### 3.2.3. Large Amplitude Oscillatory Shear (LAOS) Rheology

LAOS rheology characterizes the non-linear viscoelastic properties of materials by applying strains (or stresses) beyond the LVR. They are commonly used to mimic real-world deformation scenarios such as food processing or mastication [110]. This approach provides insights into structural transitions that occur at high stresses and strains, such as yielding and strain softening, which occur when structures within the sample are disrupted or deformed [22]. Unlike SAOS, LAOS produces a non-sinusoidal stress response that can be analyzed using software programs that perform mathematical analysis of these responses, such as Lissajous–Bowditch plots (stress *versus* strain or strain rate profiles) or Fourier transform analysis to detect higher-order harmonics. Typically, the relevant non-linear rheological parameters, as well as the fundamental harmonic modules of the material, should be reported. Similar sample preparation and measurement considerations should be used as described for SAOS analysis (Section 3.2.2).

#### 3.2.4. Uniaxial Compression Tests

Uniaxial compression tests are widely used to characterize the bulk mechanical properties of gelled foods [15]. Typically, a sample with well-defined dimensions, such as a cylinder or cube of known height and width, is placed on a measurement cell consisting of a flat plate and a probe. The probe is then made to compress the sample at a fixed rate, and the force versus distance profile is measured by the instrument. This profile is then changed into a stress versus strain profile using the known dimensions of the sample. To a first approximation, stress is the force divided by the initial cross-sectional area of the sample (σ = F/A_0_), whereas the strain is the change in length over the original length of the sample (ε = ΔL/L_0_). Stress and strain can also be calculated using other expressions that take into account changes in the dimensions of a sample during compression [111]. The initial slope gives Young’s modulus, while the first drop or discontinuity defines fracture stress and fracture strain. These metrics allow comparison of formulations and help link structure and processing to sensory firmness and breakdown.

Notably, probe geometry influences outcomes. Consequently, it is important to choose an appropriate cell (flat, round, pin, or blade) and report sample dimensions, probe type, deformation rate, final strain, temperature, and the criteria used to define yield and fracture. Furthermore, sample preparation must ensure uniformity and the absence of defects to minimize variability in the mechanical response. Air bubbles commonly introduced during emulsion gel preparation alter the rheological properties. Consequently, their presence should be reduced by altering processing conditions or by removing them (e.g., by gravity, centrifugation, or antifoaming agents).

#### 3.2.5. Texture Profile Analysis (TPA)

TPA is an instrumental protocol specifically designed to simulate the mastication process of foods by measuring the force-distance profile during a two-cycle compression-decompression test [112]. The parameters typically measured include hardness, adhesiveness, cohesiveness, springiness, gumminess, and chewiness. These measurements provide objective insights that can be correlated with the sensory perception of food products. However, some authors have stated that the TPA parameters do closely correspond to fundamental material properties or sensory properties, and can vary appreciably depending on sample size and test conditions [16]. Nevertheless, this method can still provide valuable information about food properties, and some of the problems can be overcome by using standardized testing methods. Best practices for double compression testing include clear documentation and reporting of the probe type, the maximum compression distance or strain, the test speed, and the precise definitions of each calculated parameter. Proper adherence to these protocols enhances the comparability of data across studies.

#### 3.2.6. Interpreting Rheological and Mechanical Parameters

Ideally, the various parameters obtained from dynamic shear rheology, uniaxial compression, and texture profile analysis should be related to the composition, structural organization, and interactions of the different components in emulsion gels [106,107]. However, it is often challenging to develop robust mathematical models to accurately describe the rheological and mechanical properties of emulsion gels due to their compositional and structural complexity, as well as the difficulties in measuring some of their key properties, such as particle concentration, size, and interactions, as well as polymer network structures [113,114,115]. However, some studies have shown that the rheological and textural parameters of emulsion gels can be linked to their microstructures [116,117].

Importantly, establishing structure–function relationships in emulsion gels requires integrating mechanical and rheological data with microstructural evidence. Studies on emulsion gels stabilized by plant proteins and polysaccharides have demonstrated that properties such as droplet size, network density, and biopolymer interactions modulate the shear modulus and TPA parameters [118]. In the future, more efforts should therefore be put into accurately describing the properties of emulsion gels and developing mathematical models to describe and predict their behavior [53,107,119]. The availability of these models could facilitate the rational design of emulsion gels with enhanced functional performances.

### 3.3. Thermal Analysis

Differential scanning calorimetry (DSC) is widely employed to detect and quantify thermal transitions such as melting/crystallization of lipids, denaturation of proteins, helix–coil transitions in polysaccharides, and gelatinization of starch granules [17]. This information is often useful for evaluating the performance of emulsion gels, especially for identifying the key temperatures where thermal transitions impact their gelling, melting, and viscoelastic properties. Thermogravimetric analysis (TGA) is an analytical technique used to monitor the mass loss of samples as a function of temperature, which provides insights into the thermal decomposition profiles and evaporation of volatile components such as moisture. When using these methods, it is recommended that specific operating parameters be standardized and reported, including sample pan properties, sample mass, heating/cooling rates, temperature ranges, duration of isothermal holding periods, and atmospheric conditions. Periodic calibration of DSC with appropriate thermal standards is required to ensure good data accuracy. Useful information about the thermal transitions in emulsion gels can also be obtained by using specialized analytical techniques that have temperature sweep capabilities, such as optical microscopy, scattering (Section 3.1), and rheology (Section 3.2) techniques.

### 3.4. Liquid Phase Behavior in Emulsion Gels

#### 3.4.1. Water-Holding Capacity (WHC) and Oil-Holding Capacity (OHC)

The water-holding capacity refers to the ability of a gel to retain water within its matrix under external stress, such as centrifugation [18]. Similarly, the oil-holding capacity refers to the ability of the gel to retain oil under similar conditions. In contrast, syneresis denotes the spontaneous release of water from a gel network over time, which often results from structural rearrangements. These parameters are crucial indicators of gel integrity and product stability, particularly because water or oil release may lead to undesirable textural changes. The WHC and OHC values are typically measured by subjecting a known mass of gel to centrifugation under defined conditions and quantifying the mass of water or oil released [18]. In contrast, syneresis is commonly assessed through drainage tests under gravity. The methodologies should be rigorously standardized, including conditions such as temperature, centrifugal force and duration, and storage time and temperature.

#### 3.4.2. Quantifying Liquid Phase Contribution

Valuable information about the nature of the water in emulsions and emulsion gels can be obtained using low-field nuclear magnetic resonance (LF-NMR) spectroscopy [120]. LF-NMR relaxation studies can be used to characterize the molecular mobility of the different populations of water within a sample. Typically, three populations of water are observed: T_21_ (short times), corresponding to strongly bound water; T_22_ (intermediate times), corresponding to immobilized water; and T_23_ (long times), corresponding to free water [121]. The fraction of water in each population can be assessed, which can provide valuable information about how the water is distributed within an emulsion gel. Recently, LF-NMR with multi-parametric analysis has been used to evaluate gelation, water holding, cooking losses, textural changes, and printability of surimi products, distinguishing the effects of different cryoprotectants such as D-allulose and fish protein hydrolysates [122]. LF-NMR can therefore be used to determine how formulation and processing conditions can be optimized to provide good water retention and distribution.

Fluorescence recovery after photobleaching (FRAP) is increasingly being used to quantify tracer or oil mobility inside biopolymer gels by measuring the local diffusivity within the gel network. In wax-based rapeseed oil oleogels, FRAP resolved region-specific oil diffusion and showed that dense candelilla wax networks retarded mobility, while rice bran wax spherulites permitted faster diffusion, linking microstructure to mobility [123]. A high-throughput FRAP study analyzed thousands of datasets across 18 hydrogel formulations and 7 solutes to connect mesh radius and formulation parameters to molecular diffusivity, providing valuable insights into structure–function relationships [124].

### 3.5. Investigating Gelation Dynamics

*Diffusing wave spectroscopy (DWS)*. DWS microrheology analyzes speckle intensity fluctuations from multiply scattered light to recover tracer mean-squared displacement and, via the generalized Stokes–Einstein relation, frequency-dependent moduli. It reveals hidden micro-dynamics and sol–gel kinetics in opaque samples with tiny volumes and minimal preparation [125]. In agarose hydrogels, DWS has been used to quantify the effects of biopolymer concentration and temperature on gel formation and aging [126]. When applied in food emulsion gels, this could help to rapidly screen formulations and refine processing conditions.

*X-ray photon correlation spectroscopy (XPCS)*. XPCS accesses nanoscale dynamical processes during gelation and cooking by analyzing coherent X-ray speckle fluctuations, linking structure and dynamics over sub-second to hours and nanometer to sub-micrometer length scales in out-of-equilibrium soft materials [127]. In particular, this technique has been used to isolate the roles of plasma proteins and low-density lipoproteins in the formation of heat-set egg yolk gels. It shows that viscosity is set by protein gelation and that a grainy gel microstructure arises from LDL aggregation. The gelation kinetics followed an Arrhenius-type time–temperature superposition below 75 °C but broke down above this threshold. This type of analysis results in a time–temperature phase diagram that maps the different regimes [128]. These examples suggest that XPCS would be useful for tracking droplet and protein network dynamics during the formation and application of emulsion gels.

### 3.6. Recommendations on Measurement Conditions and Sample Handling

Accurate characterization of emulsion gels requires standardized sample handling. Variations in storage time, temperature, and equilibration can significantly alter gel properties [129,130,131]. Dehydration during analysis may falsely increase the mechanical strength of a sample by increasing the polymer concentration and may alter the microstructure of samples by altering the structural organization of the different components. Using sealed chambers or cryo-techniques is therefore essential to preserve the original structure of a sample as much as possible [10,12]. Consistency in sample size and temperature control can also be used to enhance the reproducibility of rheological and thermal assessments.

## 4. Stability and Environmental Stress Testing

### 4.1. Simulated Food Matrix Stress

#### 4.1.1. Rationale

Foods vary widely in pH, ionic strength, composition, and processing history. An emulsion gel that is stable in isolation may behave differently when introduced into a complex food matrix [132]. Consequently, it is desirable to carry out stress testing under simulated food matrix conditions to provide insights into the real-world performance of emulsion gels [133].

#### 4.1.2. Relevance to Actual Storage or Processing Conditions

The stress tests should be designed to be as relevant as possible to the anticipated lifecycle of the emulsion gel [132]. Notably, if an emulsion gel is intended for use in a yogurt, its stability should be tested by incorporating it into a model yogurt system (simulating the pH, ionic strength, and milk protein network) and observing changes when the product is stored under refrigerated conditions for a period representing a typical shelf-life of yogurts [134]. If it is a component of a product that undergoes shear during filling or pumping, its resistance to mechanical stress should also be evaluated [135].

### 4.2. Long-Term Shelf and Mechanical Stability

#### 4.2.1. Monitoring Parameters over Time

To comprehensively evaluate the stability of emulsion gels, it is essential to monitor key parameters over time using a combination of analytical techniques. Visual changes serve as early indicators of physical instability. Periodic visual inspection can detect signs such as creaming, sedimentation, phase separation, oiling off, and syneresis. To illustrate, the extent of creaming can be quantified by the creaming index, calculated as the ratio of the cream layer height to the total sample height over time. Textural changes can be assessed regularly through rheological measurements such as SAOS or TPA, offering insights into processes such as softening, hardening, and structural changes during storage. Monitoring changes in the microstructure of emulsion gels during storage provides a deeper understanding of the origin of any physicochemical transformations. Techniques including CLSM and SEM can be employed at different time points to visualize droplet coalescence, aggregation, network coarsening, and complete gel breakdown, offering valuable microscopic evidence of instability mechanisms. The chemical stability of encapsulated compounds, which is particularly relevant for bioactive delivery systems, is also a critical parameter, as discussed in detail in Section 4.3. Monitoring chemical degradation complements physical assessments and ensures the functional integrity of the emulsion gel system throughout its intended shelf-life, as well as its potential health benefits after ingestion.

#### 4.2.2. Accelerated Stability Tests

The physicochemical degradation of emulsion gels may occur over long periods (weeks, months, or years). Consequently, it is often useful to establish accelerated stability tests that can speed up shelf-life prediction. These tests often subject the test sample to conditions that accelerate its tendency to break down, such as elevated temperatures, shearing, or centrifugation. However, it is important to validate these tests to be sure that they do give a true representation of the actual shelf-life of a product under normal storage conditions [136]. As an example, samples are often stored at elevated temperatures (e.g., 30–50 °C), which increases the rate of chemical degradation. The results obtained under these conditions can then be extrapolated to the stability of the product under its usual storage temperature (e.g., 4 or 25 °C) using kinetic models like the Arrhenius equation.

One caution is that the elevated temperatures should not alter the fundamental mechanism of degradation [137]. As an example, if a gel melts at the tested temperature but not at the normal storage temperature, the extrapolated results would be invalid. Validation by comparing accelerated stability test predictions with real-time stability data at normal temperatures is therefore strongly recommended.

### 4.3. Degradation Risks for Encapsulated Bioactives and Matrix Components

#### 4.3.1. Oxidation Reactions

The oxidation of lipids, bioactives, and proteins is a major concern for emulsion gels containing oxygen-sensitive compounds [138]. Lipid oxidation is especially important in emulsion gels containing polyunsaturated fatty acids. The rate and extent of lipid oxidation can be monitored by measuring primary and secondary reaction products over time [138]. Primary products can be quantified by measuring the peroxide value or conjugated diene concentration, whereas secondary products can be determined by measuring the thiobarbituric acid reactive substances (TBARSs) or anisidine value (AV). Furthermore, the loss of specific compounds or the formation of specific reaction products can be tracked over time using methods like gas chromatography (GC) or high-performance liquid chromatography (HPLC), often in combination with mass spectrometry. The rate of oxidation is influenced by several factors, including the availability of oxygen, exposure to light, temperature, and the presence of pro-oxidants, such as transition metal ions like iron and copper. It is also important to note that the large interfacial area inherent in emulsions can accelerate lipid oxidation if the interface is not adequately protected. Microfluidic studies with monodisperse emulsions found that lipid oxidation increased with decreasing droplet size, with 4.7 µm droplets oxidizing faster than 26 µm ones, which can be attributed to their larger specific surface area [139]. Consequently, it is important to understand the factors that impact oxidation in the specific emulsion gel that is being developed and to identify effective strategies to inhibit it.

#### 4.3.2. Hydrolysis Reactions

The hydrolytic cleavage of lipids and biopolymers is another major degradation pathway in emulsion gels, altering their structural integrity and adversely affecting their sensory attributes. The ester bonds found in many food ingredients, including triacylglycerols, diacylglycerols, monoacylglycerols, phospholipids, and emulsifiers, are particularly vulnerable to cleavage due to chemical or enzymatic effects. The ester bonds in the polysaccharide-based emulsifier octenyl succinic anhydride (OSA) modified starch can be cleaved by acid hydrolysis, thereby compromising its functional performance [140]. In the oil phase, interfacial lipid hydrolysis liberates free fatty acids that can generate rancid off-flavors and destabilize emulsion droplets by weakening the interfacial film [46]. The peptide bonds holding the amino acids in proteins can be hydrolyzed by proteases or under strongly acidic or alkaline conditions [141]. Similarly, the glycosidic bonds holding the sugars together in polysaccharides can be hydrolyzed by amylases or under strongly acidic or alkaline conditions [142,143]. This type of chemical degradation can weaken and disrupt biopolymer gel networks, as well as break down the protective coatings around oil droplets, thereby resulting in a loss of texture and eventual phase separation [144,145].

### 4.4. Recommendations for Designing and Reporting Stress Studies

To ensure stability, data on emulsion gels are relevant and comparable, a rigorous approach to designing and reporting stress studies is essential. The choice of stress conditions must be directly relevant to the intended application and the entire lifecycle of the emulsion gel product. A nutraceutical-loaded emulsion gel dispersed within an acidic yogurt stored at refrigerated temperatures in an opaque container will face different stresses than one that is dispersed in a neutral peanut butter spread stored at ambient temperature in a transparent container.

To properly interpret the results, the inclusion of appropriate control samples is crucial. These should ideally include the emulsion gel formulation without the encapsulated bioactive and the non-encapsulated bioactive under the same stress conditions. Following the study, reporting must be comprehensive and standardized, clearly detailing all applied stress conditions, analytical methods used, and measured parameters with statistical analysis. Moreover, robust stability assessment should extend beyond single-endpoint measurements. A kinetic analysis, with measurements at multiple time points, is strongly recommended to determine degradation rates and enable more accurate shelf-life predictions.

## 5. In Vitro Digestion Behavior of Emulsion Gels

### 5.1. Application of Gastrointestinal Simulation Models

Standardized in vitro digestion models, such as the INFOGEST protocol, have been widely reviewed and are now routinely applied by food and other scientists [27,146,147]. Since several comprehensive reviews already provide detailed coverage of gastrointestinal simulation models, we do not repeat this information here. Instead, we note that when the research question requires a closer approximation of in vivo mechanics, dynamic systems may be selected in addition to INFOGEST. Dynamic gastric models typically simulate peristaltic waves, gastric sieving, and physiologic emptying and therefore permit controlled study of how gel hardness, cohesion, and particle size distribution are influenced by gastric environments, which then impacts subsequent intestinal delivery [148,149,150,151]. Sophisticated dynamic models have also been developed to simulate other parts of the GIT. They are often computer-controlled and consist of multi-compartment models with programmed pH profiles, mucin, enzyme, and bile addition, peristaltic mixing, and a dialysis step that approximates luminal absorption [152,153]. Semi-dynamic “digestion-on-a-chip” methods have recently been developed that can be used to monitor the behavior of foods under simulated digestion conditions [154]. When colonic transformations are relevant, long-term fermentation models such as SHIME^®^ capture region-specific microbiome interactions [155].

### 5.2. Structural Effects of Emulsion Gels on Digestion

Emulsion gel structures influence the digestion and delivery of bioactive agents. The three-dimensional biopolymer networks in emulsion gels act as physical barriers that slow enzyme diffusion to O/W interfaces, with mesh sizes largely determining the rate of lipolysis [156,157]. The gradual erosion of the gel network results in a sustained, controlled release of encapsulated nutrients and bioactives. “Hard” whey protein gels with denser structures have been reported to disintegrate more slowly under gastric conditions and delay oil droplet coalescence relative to “soft” whey protein gels, which influences their lipid digestion rates [158]. Besides modulating lipid digestibility, emulsion gels can shield sensitive bioactives from gastric acidity and enzymatic degradation, thereby enhancing their stability and increasing their bioavailability [159,160,161]. However, standardized protocols to quantify gel disintegration and link network properties to nutrient release are still lacking, representing a critical frontier for future research.

### 5.3. Key Experimental Protocols and Measurements During In Vitro Digestion

#### 5.3.1. Sample Disintegration Tracking

Emulsion gels are progressively broken down as they move through the different regions of the gastrointestinal tract (GIT). Qualitative tracking by visual inspection and microscopy analysis reveals microstructure changes in digesta at different stages, which can provide qualitative and semi-quantitative information about their breakdown. Particle size and zeta potential measurements complement imaging by quantifying colloidal evolution driven by pH shifts, enzymatic action, bile salt interactions, and droplet aggregation.

#### 5.3.2. Macronutrient Digestion Assays

*Lipids*. The pH-stat remains the standard method for quantifying lipid digestion kinetics as triacylglycerols (TAGs) are hydrolyzed into monoacylglycerols (MAGs), diacylglycerols (DAGs), and free fatty acids (FFAs) [162]. More detailed information about the types and amounts of different TAGs, DAGs, MAGs, and FFAs present can be obtained using chromatography methods [163]. Emulsion gels may retard FFA release by limiting lipase access, yet in some cases, they can accelerate intestinal lipolysis by inhibiting oil droplet aggregation [164,165].

*Proteins*. When gels or emulsifiers are protein-based, digestion can be followed by sodium dodecyl sulfate-polyacrylamide gel electrophoresis (SDS-PAGE) analysis to monitor molecular-weight shifts [166]. Protein digestion can be monitored using the o-phthaldialdehyde (OPA) method to quantify free amino groups [167], or by measuring the degree of hydrolysis (DH) using the pH-stat method [168,169]. However, if a sample contains both lipids and proteins, then their digestion may both contribute to the overall pH change, due to fatty acid and amino acid release, making it difficult to determine their relative contributions. In this case, other techniques should be used. Combining supercritical fluid chromatography–mass spectrometry (SFC-MS) lipid profiling with peptidomics or amino-nitrogen assays helps decouple acidification arising from FFAs versus peptides [170].

*Starch*. For starch-containing gels, glucose oxidase–peroxidase (GOD–PAP) or reducing-sugar assays (DNS) provide rapid readouts [171,172], while HPLC with refractive-index detection (HPLC–RI) resolves saccharide distributions when hydrolysis patterns are required.

#### 5.3.3. Bioaccessibility of Encapsulated Compounds

Bioaccessibility is defined as the fraction of an ingested compound that is released from the food matrix into the gastrointestinal fluids and then becomes available for absorption [173]. After simulated intestinal digestion, the digesta is separated to isolate the micellar phase (e.g., using high-speed centrifugation and/or membrane filtration). The target compound in this phase is then quantified by HPLC, LC–MS, or spectrophotometry and reported as the micellarized fraction of the total compound present in the digesta.

Several studies have shown that emulsion gel structure and composition influence the bioaccessibility of bioactive agents. The encapsulation of curcumin within emulsion gels containing whey protein/carrageenan hydrogels in the aqueous phase has been shown to lead to a relatively high curcumin bioaccessibility (63.8%) [174]. For lipophilic bioactives, their bioaccessibility is often linked to the extent of lipid digestion, as this influences their release from the lipid phase, as well as their solubilization within the mixed micelles. In whey protein-based emulsion gels, a correlation was found between capsaicin bioaccessibility and the extent of lipid digestion, with greater lipid hydrolysis leading to greater capsaicin solubilization within the mixed micelles [158]. Measuring both lipolysis and bioaccessibility is recommended for hydrophobic bioactive substances.

#### 5.3.4. Linking Digestion to Physiological Uptake

When the objective is to evaluate transport rather than micellization alone, post-intestinal digesta can be applied under defined flow, shear, and cyclic strain to obtain permeability and uptake data with human-relevant epithelial phenotypes. Reporting shear stress, strain frequency, oxygen control, and tight-junction integrity is essential for reproducibility. For microbiome-dependent transformations, a follow-up in a simulated colon environment (such as SHIME^®^) can help capture region-resolved fermentation profiles.

### 5.4. Practical Challenges in In Vitro Digestion of Emulsion Gels

Applying standardized digestion protocols to emulsion gels presents several challenges. First, the way the gel is introduced (intact vs. minced to mimic mastication) dictates initial surface area and thus digestion kinetics, so preparation conditions must be standardized [175]. Second, the heterogeneous nature of the digesta from emulsion gels (containing undigested gel fragments, released oil, aqueous phase, and sedimented particles) can make it difficult to obtain representative samples for analysis. Phase separation and sedimentation are common issues as well [11,175]. Third, maintaining target enzyme activities and pH, then inactivating enzymes for downstream assays, can compromise the analyte. Heat inactivation risks degrading bioactives, chemical inhibitors may interfere with quantification assays, and pH adjustments can induce aggregation [27]. Fourth, if dialysis bags are employed to simulate absorption, restricted pore sizes, slow diffusion, and membrane fouling by gel components or lipids can introduce artifacts and underestimate release [176]. Moreover, components of the simulated fluids often interfere with analytical quantification of bioactives, necessitating rigorous method validation, cleanup steps, and internal standards. Finally, linking digestion to cell-culture models is further complicated by the inherent cytotoxicity of digesta, as mentioned in Section 6.1, which requires dilution or filtration that may, in turn, alter bioaccessibility [30,176].

### 5.5. Recommendations for In Vitro Digestion Studies

Ensuring high quality, reproducibility and comparability in digestion studies of emulsion gels requires detailed protocol descriptions. Researchers should describe the specific digestion model used, as well as the different components within the simulated fluids, including the source, nature, and concentration of salts, enzymes, bile salts and mucin if used. They must specify the origin and activity of the enzymes used. Details of how samples are collected and treated should also be included, such as enzyme inactivation, centrifugation, and filtration treatments.

Ideally, researchers should use standardized protocols like the INFOGEST 2.0 to facilitate cross-laboratory comparison [27], while clearly explaining and justifying any deviations. The inclusion of proper controls is indispensable. Positive controls may consist of the same emulsion without its gel network, or the free bioactive compound subjected to identical digestion conditions. Negative controls involve blank digestions without any sample to reveal background interferences, and potential toxicity of the digestion medium if following cell assays. Researchers should also explain how the bioaccessible micellar fraction was isolated and provide the equation used to calculate bioaccessibility. Where feasible, a mass balance of the bioactive compound across digesta fractions (undigested oil, mixed micelles, and sediment) should be performed to clarify its fate during digestion.

## 6. Biological Evaluation and Relevance

### 6.1. Cell-Based Models for Absorption, Bioavailability, and Local Effects

#### 6.1.1. Caco-2 Monolayers for Transport and Permeability

Caco-2 cells, derived from a human colon adenocarcinoma line, spontaneously differentiate over ~21 days into a polarized monolayer of enterocyte-like cells when grown on permeable supports [30]. These monolayers develop brush-border microvilli on the apical side and tight junctions between adjacent cells, mimicking key features of the intestinal epithelial barrier [177,178]. After in vitro digestion, the micellar fraction of an emulsion-gel digest is applied to the apical chamber, and the appearance of the bioactive in the basolateral medium is quantified over time. Monolayer integrity is crucial and routinely monitored by measuring transepithelial electrical resistance (TEER) [30,179]. Data collection typically continues until either a steady-state apparent permeability coefficient (P_app_) is reached or the TEER value remains within 10% of its initial value, thereby defining the assay endpoint.

The Caco-2 model is widely used to predict oral drug absorption and identify intestinal transport mechanisms [30]. For many compounds, the P_app_ values determined using this method correlate well with human in vivo absorption. The Caco-2 model has been applied to evaluate iron and carotenoid bioavailability from food matrices [180]. A key limitation of conventional Caco-2 monocultures is the absence of a surface-lining mucus layer, which modulates diffusion, protects against bile salts/enzymes, and shapes interactions with colloids and microbes [181]. These factors are directly relevant to assessing emulsion gel delivery systems in the gut. Recently, two complementary strategies have been developed to create mucus-bearing Caco-2 models. First, mucus formation can be induced in Caco-2 cells using an air–liquid interface (ALI) with vasoactive intestinal peptide (VIP). Recent work shows that culturing Caco-2 on Transwells under ALI conditions, with VIP added basolaterally, drives robust mucus formation (including secreted MUC2), yielding a stable, measurable mucus layer [182]. Second, Caco-2 cells can be co-cultured with mucus-producing HT-29-MTX cells to create an epithelial barrier topped by a continuous mucus layer while preserving tight junctions and transport readouts; the HT-29-MTX fraction can be tuned to adjust permeability and mucus thickness [183]. Together, these advances allow permeability assays to better reflect the physicochemical sieving and protective functions of mucus layers, thereby yielding more realistic performance tests for emulsion-gel delivery systems.

#### 6.1.2. RAW264.7 Macrophage Cell Line for Immunomodulatory Effects

The RAW264.7 murine macrophage line is widely used to evaluate the immunomodulatory potential of compounds, especially their effects on inflammation [184]. In a typical setup, cells are treated with the test sample with or without a proinflammatory stimulus like lipopolysaccharide (LPS). Key endpoints include nitric oxide production measured by the Griess assay, phagocytic activity quantified by the uptake of fluorescent particles or labeled bacteria, and cytokine profiling of both pro-inflammatory mediators (TNF-α, IL-6, IL-1β) and anti-inflammatory cytokines (IL-10) using ELISA or multiplex assays. Gene expression analysis of inflammatory markers by qPCR further elucidates underlying mechanisms [32]. This model helps identify components that may have local immunomodulatory effects in the gut or systemic effects if absorbed.

#### 6.1.3. Co-Culture Models (e.g., Caco-2/RAW264.7; Caco-2/HT29-MTX)

Co-culture systems can provide more physiologically relevant in vitro gut models by combining intestinal epithelial cells with other relevant cell types:

Caco-2/RAW264.7 co-cultured models typically involve growing Caco-2 cells on the apical side of a Transwell insert and RAW264.7 macrophages in the basolateral compartment [185]. This setup allows for the study of interactions between the intestinal epithelium and immune cells, such as the epithelial response to a stimulus, subsequent signaling to macrophages, and the resulting immune activation or modulation. It is often used to assess barrier function changes, transepithelial signaling, and inflammatory responses [186]. Studies utilized a Caco-2/RAW264.7 co-culture model to simultaneously evaluate barrier integrity and macrophage-mediated inflammatory responses to stimuli or digested bioactives.

The Caco-2/HT29-MTX co-culture integrates mucus-secreting goblet cells with absorptive enterocytes to recreate the cellular complexity and mucus layer of the small-intestinal epithelium, thereby enabling the formation of a continuous, adherent mucus barrier and intercellular pore architecture more representative of in vivo tissue [187]. This model yields tighter control over paracellular permeability (e.g., reduced lipophilic compound flux but closer human-like pore radius), improves prediction of nutrient and drug uptake, such as iron bioavailability, and enhances correlation with in vivo absorption data [187,188,189]. In some cases, this model has been combined with Raji B cells that simulate M-cells, thereby more accurately reflecting the complexity of the human gastrointestinal tract lining [190].

### 6.2. Animal and Human Trials

#### 6.2.1. Animal Models

Because in vitro digestion models cannot capture the full gastrointestinal complexity [27], animal studies are used to examine the fate of emulsion gels and the absorption, metabolism, and activity of encapsulated bioactives. Mice and rats are often selected for cost, short lifecycles, and genetic tools, while pigs are increasingly used for their human-like gastrointestinal anatomy, physiology, and microbiota [33]. In line with the World Medical Association Declaration of Helsinki, animal testing typically precedes human trials [191].

The dosing strategy should be selected according to the objective and duration of the in vivo study. Oral gavage of an emulsion gel allows precise administration of a single dose for pharmacokinetic profiling, while mixing an emulsion gel into chow is more suitable for longer-term efficacy trials [192,193]. The bioactive doses used are typically scaled allometrically from in vitro efficacy data or derived from existing literature. During a study, serial blood samples (collected from the tail or saphenous vein) enable the construction of plasma concentration-time curves for pharmacokinetic and bioavailability analysis. The maximum concentration (C_max_), time to reach the maximum concentration (T_max_), area under the curve (AUC) and half-life of the bioactive substance can be determined [194]. Urine and feces are collected for excretion and mass-balance analysis. At necropsy, target organs and luminal contents are sampled to assess tissue distribution and in vivo digestion.

All work should follow Institutional Animal Care and Use Committee oversight and the 3Rs principles of Replacement, Reduction, and Refinement [195]. Transparent reporting following Animal Research: Reporting of In Vivo Experiments (ARRIVE) guidelines is essential, including study design, sample-size justification, randomization, blinding, animal characteristics, housing, procedures, and statistical methods.

#### 6.2.2. Human Clinical Trials

Human clinical trials are the definitive means to establish the bioavailability, efficacy, and safety of food-grade emulsion gel delivery systems [196]. Randomized controlled trials (RCTs) are considered to be the gold standard for these kinds of tests. RCTs may use either parallel or crossover designs; in crossover studies, each participant receives all treatments in a randomized sequence with a sufficient washout period, which can reduce inter-individual variability and improve statistical efficiency relative to parallel designs [196,197,198]. Single-dose studies are typically conducted to characterize the pharmacokinetics of bioactive agents (such as the bioavailability, C_max_, T_max_, and half-life), while repeated-dose supplementation trials assess efficacy by measuring changes in relevant biomarkers or health outcomes over weeks to months [196,197]. Serial blood collections allow calculation of standard pharmacokinetic parameters. When appropriate, urine and feces sampling can further refine pharmacokinetic profiling. Targeted assays can quantify a range of physiological and biochemical markers, including plasma levels, lipid profiles, inflammatory mediators, and oxidative-stress indices. RCTs can also be designed to assess changes in gut microbiota or to provide information about changes in human performance, such as attention, cognition, or mood.

All human research must be approved by an Institutional Review Board or an independent ethics committee [199]. Participants must provide informed consent after full disclosure of the study aims, procedures, risks, and benefits, in accordance with the Declaration of Helsinki principles [191]. Study designs should minimize risk, ensure fair subject selection, and protect privacy. Reporting should follow CONSORT, including details on randomization, blinding, participant flow and baseline characteristics, intervention composition and delivery, outcome definitions, sample-size justification, and statistical methods [200]. Reproducibility depends on complete documentation of the emulsion-gel formulation, including source materials, processing steps, structure, physicochemical properties, dosage form, schedule, administration conditions, and compliance monitoring. Although many clinical trials of structured emulsions focus on pharmaceutical systems, there remains a need for well-characterized trials on food-grade emulsion gels that integrate pharmacokinetic and biomarker endpoints to guide functional food applications.

### 6.3. Linking Emulsion Gel Properties to In Vivo Behavior

A key goal of emulsion gel research is to link their compositions, structures, and physicochemical properties to their in vivo performance. The gastrointestinal residence time of a food depends on gel strength, erosion rate, and particle size after initial disintegration. Harder, more robust gels often empty from the stomach more slowly or in larger fragments, which can prolong digestion and nutrient or bioactive release. The mucoadhesive properties of the emulsion gel may also affect how long it remains in the GI tract. Certain biopolymers used to formulate emulsion gels, notably pectin, exhibit mucoadhesive behavior. The methyl ester and free carboxyl groups of pectin form hydrophobic and hydrogen bonds with mucin glycoproteins. These interactions increase the viscosity of pectin–mucin mixtures and prolong residence times in the gastrointestinal mucus layer [201]. In general, polymer charge, hydrophobicity, hydrophilicity, and chain flexibility all influence mucoadhesion by altering the electrostatic, hydrophobic, and hydrogen bonding interactions.

The in vivo release profiles of nutrients and bioactive agents from emulsion gels depend on several factors. Smaller droplets typically provide more specific surface area for lipase, speeding lipid digestion and bioactive release. Gel networks that inhibit droplet coalescence in the mouth and stomach may increase lipid digestion in the small intestine because of the higher specific surface area. However, dense gel networks may slow the enzyme penetration and matrix erosion, thereby inhibiting lipid digestion and bioactive release. Strong interactions between droplets and the gel matrix can also alter the release kinetics. Finally, the digestibility of gelling agents and emulsifiers determines how quickly the encapsulated compounds are released. Pickering emulsions, with their particle-stabilized interfaces, often offer sustained release and protect bioactives. Specifically, microgel composition has been shown to modulate α-amylase-triggered release of encapsulated droplets, which in turn altered their lubrication behavior under enzymatic hydrolysis [202].

### 6.4. Challenges and Limitations of Translating In Vitro to In Vivo Outcomes

In vitro models capture only a fraction of the complexity of the human gastrointestinal tract. They cannot fully replicate the continuous mucus layer, the diverse and metabolically active microbiota, dynamic peristalsis and mixing patterns, or neurohormonal feedback mechanisms that all influence nutrient digestion and absorption [203]. Interactions with other dietary components in mixed meals further complicate nutrient release and uptake in ways that simplified in vitro systems cannot accurately model or predict.

Another key distinction that is important to be aware of is between bioaccessibility and bioavailability [204]. In vitro digestion primarily measures bioaccessibility, which is the fraction of a compound is solubilized in the simulated gastrointestinal fluids in a form that is suitable for absorption, In contrast, bioavailability is a measure of the fraction of ingested bioactive agents that reaches the systemic circulation, which depends on absorption into enterocytes, potential efflux back into the lumen, and metabolism in the intestine and liver [173]. Caco-2 assays help bridge this gap but still fall short of accurately capturing intracellular metabolism and transporter-mediated efflux steps.

Human populations exhibit wide inter-individual variability in gut physiology, enzyme activities, microbiota composition, genetics, diet and lifestyle [205]. None of these factors are usually included in standardized in vitro protocols, making it difficult to predict how different people will process the same emulsion gel. Similarly, animal models introduce their own species-specific differences in GI anatomy, transit time, enzyme expression and microbiota, which can limit the direct extrapolation of their findings to humans.

Practical challenges also undermine the reliability of in vitro data. As discussed in Section 5.4, issues such as digesta toxicity to cell lines, the need to simulate mastication for solid gels, and sampling errors from heterogeneous digesta can all skew results. Moreover, the correlation between Caco-2 permeability and human bioavailability is compound-dependent: it may be strong for some nutrients (e.g., iron in certain contexts) but poor for others (e.g., carotenoids). Such variability underscores the importance of validating each in vitro method against appropriate in vivo or clinical data before concluding.

## 7. Applications

**Figure 5:**
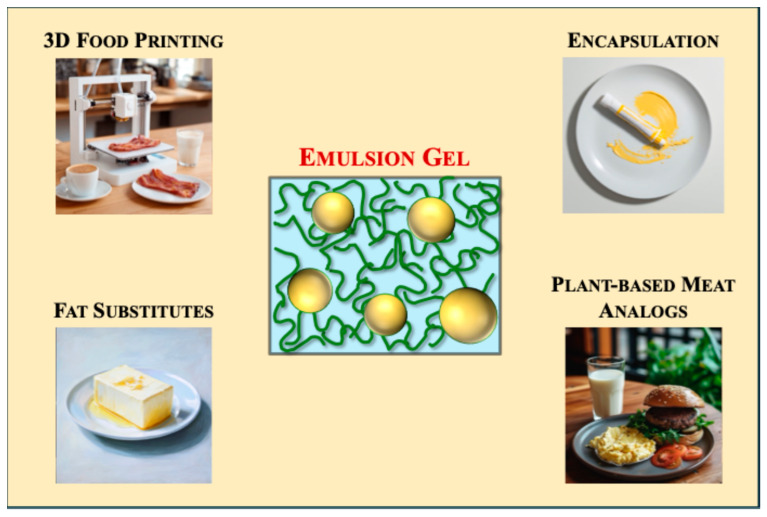
Emulsion gels have a broad range of potential applications in the food industry. Images created by Midjourney (V7, www.discord.com, accessed on 12 February 2026).

There is a broad range of potential applications of emulsion gels in the food industry, some of which are summarized schematically in Figure 5 and discussed briefly in the following subsections.

### 7.1. Encapsulation of Lipophilic Nutraceuticals

Emulsion gels can be used to protect sensitive bioactives from degradation, as well as to modulate their retention and release profiles. Researchers have engineered high-internal-phase gelatin-based emulsion gels containing up to 80% sunflower oil loaded with β-carotene, achieving retention rates of 90% after 27 days of storage, compared to only 8% retention in bulk oil, thereby underscoring the protective effects of the hydrogel network [206,207]. In another study, researchers showed that edible inks containing β-carotene could be created by optimizing the composition and structure of chitosan/OSA-β-cyclodextrin-stabilized emulsion gels [208]. Moreover, the bioaccessibility of the β-carotene in these emulsion gels was relatively high (41.9%), as determined using an in vitro gastrointestinal tract study. In another in vitro study, it was shown that whey protein/κ-carrageenan composite emulsion gels could be formulated that had a relatively high curcumin bioaccessibility (63.8%), which was mainly attributed to the small size of the oil droplets in these systems produced by HPH [174].

Collectively, these studies highlight the promise of food-grade emulsion gels as versatile delivery platforms for improving the stability, release profile, and efficacy of lipophilic nutraceuticals in complex food matrices.

### 7.2. Fat Substitutes

Emulsion gels have also been investigated for their potential to be used as healthy fat substitutes in foods because they can be created with semi-solid textures that somewhat resemble those of semi-solid fats. Researchers have substituted more than 50% of animal fat with emulsion gels, while still maintaining a desirable texture and sensory attributes [209]. In another study, the pork fat in sausages was partially or completely replaced with emulsion gels formulated from plant oils, proteins, and polysaccharides, which resulted in a lower fat content while maintaining good texture, cooking yield, and consumer appeal [210,211]. Similarly, substituting pork fat with xylo-oligosaccharide-rich emulsion gels was found to lower the lipid content by around 30% and improve the nutritional profile, while still retaining the desired physicochemical qualities [212]. In summary, emulsion gels have considerable potential for application as fat replacers in foods because they can provide semi-solid textures while increasing the level of unsaturated fatty acids and lowering the level of saturated ones, thereby enhancing their nutritional attributes.

### 7.3. Probiotic Encapsulation and Protection

Probiotics are living microorganisms that exhibit health benefits when consumed in sufficient quantities [213]. However, they are often inactivated when exposed to food processing conditions, such as heating or shearing, or when they pass through the human digestive tract due to the highly acidic nature of the gastric fluids, as well as the presence of antimicrobial bile salts and digestive enzymes within the small intestine [214,215]. Probiotic encapsulation within emulsion gels may be able to partially mitigate these problems. Microorganisms have hydrophilic surfaces and so they are typically encapsulated within the hydrogel matrix in the aqueous phase of emulsion-filled gels.

Several studies have already demonstrated the potential of emulsion gels to protect probiotics. For instance, encapsulation of *Lactobacillus plantarum* within whey protein–EGCG conjugate/gellan gum emulsion gels enhanced their colon-controlled delivery and retained over 85% of their viability, which was mainly attributed to the formation of a dense, homogeneous double-network hydrogel in the aqueous phase [216]. In another study, *Lactobacillus plantarum 299v* was encapsulated in emulsion gels containing xanthan and guar gums in the aqueous phase [217]. The probiotics in these emulsion gels were shown to exhibit good resistance to storage, chilling, freezing, and cryopreservation, as well as sustained probiotic release under simulated colonic conditions. Probiotics can also be trapped within the internal aqueous phase of water-in-oil-in-water (W/O/W) emulsion gels to protect them from their environment. *Lacticaseibacillus rhamnosus* and *Lactobacillus gasseri* have been co-encapsulated with fructooligosaccharides in basil seed gum-stabilized W/O/W emulsions, which were found to retain over 90% probiotic viability during heat processing, gastrointestinal simulation, and 28-day storage [218]. High-internal-phase emulsion gels have also been shown to protect encapsulated probiotics (*Lactobacillus plantarum*) from degradation when they pass through the gastrointestinal tract using an in vivo zebrafish model [219].

### 7.4. Plant-Based Food Analogs

Emulsion gels are increasingly being used as ingredients to formulate plant-based analogs of traditional animal-derived products, such as meat, seafood, egg, and cheese. In particular, the semi-solid nature of these products can be useful for simulating the textural attributes of these products. Researchers have shown that emulsion gels composed of soybean oil droplets embedded in potato protein hydrogels can be used to simulate the properties of lean meat [220]. Moreover, HIPE gels formulated from soybean oil, soy protein, and gelling hydrocolloids have been successfully used to mimic the properties of beef adipose tissue [221,222]. In another study, plant-based egg yolk was produced that consisted of olive oil droplets fortified with β-carotene and vitamin D embedded within a hydrogel phase consisting of β-glucan and potato protein [223]. These emulsion gels were shown to effectively mimic the appearance, texture, cookability, and nutritional profile of egg yolk. Emulsion gels are also being used to create dairy analogs. Pickering emulsion gels created from soybean oil, microfibrillated cellulose, soy protein, and corn starch have been shown to closely reproduce the color, texture, and meltability of sliced cheese [224]. Similarly, composite starch/fermented protein emulsion gels have been shown to have mechanical properties that closely match those of cheese, but their melting response still remains to be optimized [225]. Emulsion gels prepared from whole faba bean flour have been used to formulate yogurt analogs that have gel strengths and water-holding capacities somewhat similar to those of dairy yogurt [226]. In another study, researchers showed that high-pressure processing of plant oils (from sunflower) and proteins (from mung bean, chickpea, pea, lentil, or faba bean) led to the formation of emulsion gels with viscoelastic properties similar to those of commercial Greek yogurt [227].

### 7.5. Edible Inks in 3D Food Printing

Emulsion gels have also been investigated for their potential application as edible inks in 3D food printing [228]. Edible inks must be able to flow through a nozzle when put under pressure, but then rapidly set once they have been deposited on the printing platform, and then retain their shape and properties over time. Edible inks can be designed to exhibit these attributes by optimizing their compositions and structures, especially the size and concentration of oil droplets, as well as the type and amount of gelling agents used. Moreover, emulsion gels can be formulated to contain a wide range of different functional and nutritional ingredients, including colors, flavors, vitamins, minerals, and nutraceuticals. Consequently, they can be used to create food products whose sensory attributes and nutritional profiles can be personalized for specific individuals or groups. In particular, emulsion gels stabilized by pea protein particles and flaxseed have been successfully used to formulate edible inks with good rheological and printing properties [228]. When the emulsion gel formulation was optimized, the printed objects had smooth surfaces and precise shapes. In another study, researchers were able to create edible inks from emulsion gels formulated from sunflower oil, whey protein, and asparagus fiber [229]. They showed that the rheological properties of these edible inks could be modulated by varying the oil or fiber concentration, which allowed their printability to be optimized. Recently, a population-balance model has been developed that can relate the composition and structure of emulsion gels to their rheological properties [230]. This type of model may be useful for optimizing the functional performance of edible inks for different 3D food printing applications.

## 8. Conclusions

This review provides a workflow-oriented framework for advancing food-grade emulsion gels as nutrient delivery systems by linking formulation and processing design with physicochemical characterization, application-relevant stability testing, digestion assessment, biological evaluation, and transparent reporting within a single integrated approach. In this way, it moves beyond a descriptive summary of emulsion gel systems and offers practical guidance for generating more rigorous, reproducible, and comparable studies. Key priorities for the field include tighter optimization, control, and reporting of formulation and processing parameters; multi-scale structural and physicochemical characterization; and broader adoption of standardized in vitro digestion models, such as INFOGEST 2.0, with suitable adaptation for gel-based matrices. Where appropriate, biological validation should follow a tiered strategy, beginning with cell-based models and progressing through suitable animal studies to human trials. Looking ahead, the field will likely evolve toward more application-specific and scalable formulations, testing under realistic food processing and storage conditions, more physiologically relevant in vitro and in vivo models, and greater harmonization of experimental and reporting practices. These advances should strengthen translation from laboratory systems to commercial food products, improve reproducibility and comparability across studies, and support data-driven design of next-generation functional foods.

## Figures and Tables

**Table 1:** Summary of key physicochemical characterization techniques for emulsion gels.

Technique	Key Parameters Measured	Typical Information Obtained	Potential Pitfalls	Reference	
Structural					
Optical microscopy	Droplet size, morphology, general structure	Basic visualization of emulsion and gel structure	Resolution limited; sample thickness		
Confocal laser scanning microscopy (CLSM)	3D microstructure, droplet size/distribution, network morphology, phase colocalization	Detailed visualization of hydrated structures, interactions between phases	Dye selection and concentration, photobleaching, artifacts from dyes, penetration depth	[10]	
Scanning electron microscopy (SEM)	Surface topography, network porosity (dried samples)	High-resolution imaging of dehydrated or cryo-fractured surfaces	Sample preparation (dehydration, fixation, coating method and material); artifacts from drying	[11]	
Cryo-SEM	Preserved hydrated microstructure, internal network, droplet morphology	More native-like structure visualization compared to conventional SEM	Freezing protocol (rate, cryoprotectants), fracture method, coating; ice crystal damage	[12]	
Transmission electron microscopy (TEM)	Nanoscale details, interfacial layers, internal droplet structure, particle morphology	Ultra-high resolution of internal features	Sample preparation (staining, sectioning, embedding), beam damage; artifacts from staining/fixation.	[11]	
Light scattering (DLS/SLS)	Droplet/particle size distribution (DLS), PDI (DLS), MW, Rg (SLS)	Characterization of pre-gelled phase	Refractive indices, viscosity of medium, scattering angle; multiple scattering in concentrated systems	[13,14]	
Small-angle X-ray scattering and small-angle neutron scattering (SAXS/SANS)	Nanoscale structure (1–100 nm), mesh size, interfacial thickness, particle shape	Quantitative information on molecular organization and network parameters	Complex data modeling, contrast requirements (SANS); sample concentration		
Rheological & mechanical					
Small-amplitude oscillatory shear (SAOS)	Storage modulus (G′), loss modulus (G″), Tanδ, complex viscosity (η*)	Linear viscoelastic properties, gel strength, gelation kinetics, frequency dependence of network structure	Geometry; sample loading, slip, edge effects		
Large amplitude oscillatory shear (LAOS)	Non-linear moduli, Lissajous plots, harmonic intensities	Non-linear viscoelastic behavior, structural breakdown under large deformation	Strain amplitude range, frequency; complex data analysis		
Texture profile analysis (TPA)	Young′s modulus, fracture stress, fracture strain, yield point; hardness, cohesiveness, springiness, adhesiveness, gumminess, chewiness	Bulk mechanical strength, stiffness, deformability, brittleness; instrumental correlation to sensory texture attributes	Sample dimensions and uniformity, deformation rate, temperature; probe type and size, compression settings	[15,16]	
Thermal & water-holding					
Differential scanning calorimetry (DSC)	Transition temperatures (Tm, Tg, Tc), enthalpies (ΔH)	Phase transitions	Pan type; baseline subtraction, calibration	[17]	
Thermogravimetric analysis (TGA)	Mass loss vs. Temperature	Thermal stability, decomposition profiles, water content	Ensure full decomposition		
Water-holding capacity (WHC)	Water retained (%	Gel’s ability to hold water under stress	Centrifugation force and time, temperature	[18]	

*Note:* The table was prepared by the authors as a concise synthesis of the main methods discussed in Section 3, based on the representative methodological references summarized in Table 2.

**Table 2:** Foundational and practical studies guiding experimental methods in food-grade emulsion gel research.

Experimental Technique	Key Summary	Reference
Confocal laser scanning microscopy (CLSM)	Detailed overview of CLSM principles for food microstructure, covering optical sectioning, dye selection, image processing, and artifact avoidance.	[10]
Scanning electron microscopy (SEM & Cryo-SEM)	Guidance on preserving native gel/emulsion structure in SEM, emphasizing fixation methods and cryogenic imaging to prevent dehydration artifacts.	[11]
	Protocol for advanced cryo-SEM of protein hydrogels using high-pressure freezing, pFIB milling, and low-dose imaging to minimize damage.	[12]
Transmission electron microscopy (TEM & cryo-TEM)	Review of TEM techniques for nanofoods, stressing the importance of rapid freezing and low-dose imaging to preserve hydrated structures.	[11]
	Comparison of conventional vs. cryo-TEM for Pickering emulsions, detailing protocols to avoid beam damage and ice-crystal artifacts.	[19]
Light scattering (SLS/DLS)	Introduction of the Zimm plot for extracting molecular weight and size from static scattering data	[20]
	Comprehensive guide to photon correlation spectroscopy theory and instrumentation for DLS.	[13]
	Practical DLS protocols for diverse biological and colloidal systems, addressing measurement consistency.	[14]
Oscillatory shear rheology (SAOS/LAOS)	Comprehensive guide to dynamic oscillatory shear techniques for assessing linear viscoelasticity and monitoring gelation in food and soft matter systems	[21]
	Comprehensive review of LAOS methodology, emphasizing Lissajous–Bowditch plots and frameworks for nonlinear viscoelastic interpretation in complex fluids	[22]
	Survey of LAOS applications in foods: linking nonlinear viscoelastic parameters to microstructure, processing behaviors, and sensory/oral-processing attributes.	[23]
Texture profile analysis (TPA)	Reviews the evolution and application of TPA to gelled foods: covers key instruments, testing conditions, parameter definitions, and reproducibility/sensory correlations.	[15]
	Critically evaluates classical TPA parameters and advocates adopting standardized mechanical tests of intensive properties for reliable texture measurement	[16]
Differential scanning calorimetry (DSC) & thermal-gravimetric analysis (TGA)	Overview of DSC principles and their applications for detecting protein denaturation, starch gelatinization, fat polymorphism, and water transitions in food gels, with guidance on sample preparation and thermogram interpretation.	[17]
	Demonstration of combined DSC and TGA to characterize emulsion gel thermal behavior and stability.	[24]
Water-holding capacity (WHC)	Microcentrifuge-based procedure for quantifying gel water retention, detailing centrifugal force (g), run time, sample dimensions, and held-water calculations.	[18]
	Empirical study linking gel syneresis rates to rheological properties under controlled conditions. Presents underlying theory: gel network contraction generates a pressure that expels water and relates this to gel rigidity.	[25]
In vitro static digestion (INFOGEST protocol)	Standardized static digestion protocol with defined fluid compositions, phases, and endpoints for reproducible in vitro trials	[26]
	An updated “INFOGEST 2.0” protocol reflecting 5 years of improvements. Refines certain steps. Includes a troubleshooting section for common issues. Emphasizes sample handling for gels/emulsions. Provides representative results and notes on variability.	[27]
Bioaccessibility (Micelle separation & quantification)	Detailed protocol for assessing carotenoid bioaccessibility via INFOGEST digestion, micelle isolation by high-speed centrifugation and membrane filtration, solvent extraction, and HPLC quantification, emphasizing key steps like phase separation and use of controls.	[28]
	Examines how micelle separation conditions influence carotenoid bioaccessibility measurements, demonstrates the need for matrix-specific protocols, and develops a predictive model linking intestinal digesta properties to optimal centrifugation parameters.	[29]
Cell-based bioactivity (Caco-2 absorption)	Stepwise Caco-2 monolayer assay protocol covering seeding density, medium composition, transepithelial resistance (TEER) integrity checks, timed apical dosing and basolateral sampling for quantification, with controls to distinguish active versus passive transport.	[30]
	Pioneering study using Caco-2 cells to measure iron uptake as an indicator of bioavailability from foods. Details controlling cell culture conditions to not confound results.	[31]
Cell-based bioactivity (RAW264.7 inflammation model)	Comprehensive RAW264.7 anti-inflammatory assay protocol covering cell culture and LPS stimulation, MTT and CCK-8 viability assays, nitric oxide quantification via the Griess reaction, ELISAs for TNF-α, IL-1β and IL-6, quantitative real-time PCR, Western blotting, and key troubleshooting tips	[32]
In vivo studies (animal/human bioavailability)	Template for animal trials of β-carotene emulsion gels, detailing dosing schedules, blood sampling, and plasma analysis.	[33]

## Data Availability

No new data were created or analyzed in this study.
